# From Muscle–Bone Concept to the ArthroMyoFascial Complex: A Pragmatic Anatomical Concept for Physiotherapy and Manual Therapy

**DOI:** 10.3390/life14070799

**Published:** 2024-06-25

**Authors:** Karl Noten, Robbert van Amstel

**Affiliations:** 1 Fysio Science Department, Fysio Physics Group, 3401 IJsselstein, The Netherlands; karl.noten@fysiophysics.nl; 2 Department of Human Movement Sciences, Faculty of Behavioural and Movement Sciences, Amsterdam Movement Sciences, Vrije Universiteit Amsterdam, 1081 Amsterdam, The Netherlands

**Keywords:** fascia, skeletal muscles, joints, anatomy, physical therapy specialty

## Abstract

Background: In physiotherapy, the classic muscle–bone concept is used to translate basic and clinical anatomy. By defining the anatomical structures from superficial to deeper layers which frame the ArthroMyoFascial complex, our aim is to offer clinicians a comprehensive concept of within the muscle–bone concept. Method: This study is a narrative review and ultrasound observation. Results: Based on the literature and ultrasound skeletonization, the ArthroMyoFascial complex is defined. This model clarifies fascial continuity at the joint level, describing anatomical structures from skin to deeper layers, including superficial fascia, deep fascia, myofascia including skeletal muscle fibers, and arthrofascia all connected via connective tissue linkages. This model enhances the understanding of the muscle–bone concept within the larger ArthroMyoFascial complex. Conclusion: The ArthroMyoFascial complex consists of multiple anatomical structures from superficial to deeper layers, namely the skin, superficial fascia, deep fascia, myofascia including muscle fibers, and arthrofascia, all linked within a connective tissue matrix. This model indicates that it is a force-transmitting system between the skin and the bone. This information is crucial for manual therapists, including physiotherapists, osteopaths, chiropractors, and massage therapists, as they all work with fascial tissues within the musculoskeletal domain. Understanding fascia within the muscle–bone concept enhances clinical practice, aiding in therapeutic testing, treatment, reporting, and multidisciplinary communication, which is vital for musculoskeletal and orthopedic rehabilitation.

## 1. Introduction

Physiotherapists and manual therapists use various interventions that aim to improve muscle and joint functions. It is worth noting that physiotherapists, osteopaths, chiropractors, and massage therapists fall under the category of manual therapists. Manual therapists, in general, emphasize the evaluation and treatment of muscle flexibility and joint mobility that causes muscle-joint dysfunction, and pain [1,2]. Fascia tissue manipulations as treatment interventions have gained increased attention [3,4,5,6]. Fascia tissue manipulations encompass methods such as soft tissue mobilizations, application of elastic tape, myofascial release techniques, and joint high-velocity thrust manipulations and mobilizations [7]. Applying force during fascia tissue manipulation is expected to stress and strain the fascial tissues underlying the skin, which is thereby expected to modulate specific parts of anatomical structures surrounding a muscle and/or joint [8].

A comprehensive terminology was suggested at the International Fascia Research Congress regarding the classification of fascial tissues [9], which has been updated [10]. The integration of fascia primarily focuses on the superficial fascia, deep fascia, and myofascia, largely due to their origins in massage therapy, which predominantly targets muscle treatments. In physiotherapy, the classic muscle–bone concept is used to translate basic and clinical anatomy [11,12]. This concept is based on the principles that muscle and bone are genetically, molecularly, and mechanically interconnected [12].

Considering that muscles are epimuscularly attached to bones, joint capsules, and ligaments [13], and that joint capsules and ligaments are also classified as fascia [9], the muscle–bone concept is no longer adequate. This is further emphasized by the myofascial unit concept [14]. The concept of the myofascial unit elucidates that in numerous motor tasks, active muscle engagement interacts with periods of passive muscle behavior [15]. In addition, it offers a potential explanation of how muscles interact with bones, not only through the muscle–tendon unit, but also through the extramuscular connective tissue linkages when the muscle actively contracts [16]. It is suggested that extramuscular anatomical structures like joint capsules and ligaments (referred to as ‘arthrofascia’), as well as deep fascia and even superficial fascia, may have the capacity to influence skeletal muscle function, thereby impacting joint mobility [13]. Therefore, it is hypothesized that these anatomical structures collectively contribute to optimal motor control, collaborating synergistically, to optimize functional movements in the most effective manner [13,15]. Understanding the complex interplay between fascia, muscle, bone, and a joint during functional movements starts by integrating basic knowledge of fascia anatomy in the muscle–bone concept.

Since the muscle–bone concept is integrated in physiotherapy education courses, clinicians consequently continue to face challenges in defining fascia as an anatomical structure, essential for clinical applications and peer communication. Therefore, this narrative review aims to comprehensively describe the linkages from the skin to bone at the level of a joint, including anatomical structures such as skeletal muscles, fascial tissues, and neurovascular tracts. By defining the anatomical structures from superficial to deeper layers which frame the ArthroMyoFascial complex, our aim is to offer clinicians a comprehensive classification of fascial tissues within the muscle–bone concept. The significance of this work lies in enhancing the clinical concept of fascial tissues, which is crucial for therapeutic testing, treatment, reporting, and multidisciplinary communication, important for comprehending musculoskeletal and orthopedic rehabilitation.

## 2. Anatomical Description of Fascia

First, this review provides background information to describe the basics of fascia, classified with the identifier codes (IDs) established by the International Federation of Associations of Anatomists (IFAA) through the Anatomical Terminology. Secondly, we briefly explain and classify each anatomical fascial tissues and its linkages within this ArthroMyoFascial complex. Thirdly, we analyze the proof of concept through quantitative analyses of ultrasound images using skeletonization. While detailed explanations are omitted, this information is deemed essential for evaluating and discussing the proposed model.

### 2.1. Fascia Basics

Fascia is a specialized connective tissue sheath and is most often misused as a synonym for connective tissue, simply because it is not known that it is a general name for various phenotypic fibrous anatomical structures like the stratum membranosum, deep fascia, epi-peri-endomysium, capsules, and ligaments (IFAA ID: A04.0.00.031), which are interconnected (Table 1) [9,10]. In other words, it is a fascial network that consists of different fascial tissues (i.e., fasciae) [17]. A fascia is a sheath of connective tissue that forms beneath the skin to attach, enclose, and separate not only muscles but also bones, nerves, blood vessels, and organs [10]. Each fascia within this network consists of a unique extracellular matrix encapsulating fibroblasts, myofibroblasts, and fasciacytes [18,19] that determines the fascial biomechanical content. This biomechanical content is expressed in viscoelasticity, which determines the time-dependent stiffness and thereby the resistance to stress and strain. Considering these elements, this anatomical structure could be classified into (1) a fascia, (2) fasciae (multiple fascia), and (3) the fascial system [9,10,17], connected from the skin to the bone within an ArthroMyoFascial complex. 

### 2.2. Superficial Fascia

The skin (IFAA ID: A16.0.00.002) consists of epithelium and is composed of three layers: the epidermis, dermis, and hypodermis [20]. The layers known as the epidermis, dermis, and hypodermis are not categorized as fascia. Nevertheless, the stratum membranosum (IFAA ID: A16.0.03.005) present within the hypodermis is commonly recognized as a type of fascia [9]. The stratum membranosum is also known as the superficial fascia [21]. The superficial fascia (IFAA ID: A04.0.00.031) is a connective tissue network and varies in thickness [22]. The superficial fascia is a connective tissue network with a membrane that separates the superficial adipose tissue from the deep adipose tissues. The superficial fascia membrane is superiorly attached to the dermis via the retinacula cutis superior and inferiorly to the deep fascia via the retinacula cutis inferior (IFAA ID: A16.0.00.005) [17,23]. For functional purposes, at some locations, this linkage is more firm via skin ligaments (Cooper’s ligaments), and at other locations, it is filmier and loose [24]. In addition, the superficial fascia is a densely innervated structure. Research revealed extensive innervation within the superficial fascia, emphasizing its significance in thermoregulation, sensation, and pain perception, which could enhance the understanding of factors impacting superficial fascia sensitivity [25].

### 2.3. Deep Fascia and Myofascia Including Muscle Fibers

Deep fascia (IFAA ID: A04.0.00.031) also known as the fascia generalis or fascia profunda is a strong and dense sheath of connective tissue [26]. It forms a continuous sheath that covers muscles, bones, nerves, and blood vessels, helping to compartmentalize and protect these structures [27]. 

For example, well-known deep fascia layers are the thoracolumbar fascia (IFAA ID: A04.3.02.501) [28] and fascia lata (IFAA ID: A04.7.03.002) [29]. The fascia lata is a dense connective tissue that surrounds the thigh muscles. It is the outermost layer of fascia in the thigh and extends from the hip to the knee [29]. Within the fascia lata, there is a thick band of fibrous tissue known as the iliotibial tract (IFAA ID: A04.7.03.003). It runs along the outside of the thigh, extending from the pelvis (specifically the iliac crest) down to the knee, where it attaches to the tibia and the fibula [29]. The thoracolumbar fascia is a specific deep fascia located in the lower back region, covering the muscles of the lumbar and thoracic spine. It consists of several layers of fibrous tissue and serves as a critical attachment site for various muscles, including the latissimus dorsi, gluteus, and transverse abdominis [30], facilitating force transmission among them [31] via the so-called lateral raphe [32,33]. Without further specific expansion, it is evident that deep fascia is often named based on its anatomical location. Examples include the brachialis intermuscular septa (IFAA ID: A04.6.03.006) found in the upper arm (brachium), the popliteal fascia (IFAA ID: A01.2.08.013) located at the back of the knee (poples), and the fascia plantaris (IFAA ID: A04.7.03.031) at the sole of the foot (plantar). These names help to identify the precise areas where these fascial structures are prominent.

Skeletal muscles (IFAA ID: A04.0.00.000) consist of a complex three-dimensional connective tissue scaffold resembling a honeycomb structure [9]. This myofascial scaffold includes several interconnected layers: the epimysium, perimysium, endomysium, and tendon [34]. The epimysium (IFAA ID: A04.0.00.041) is the outermost layer that envelops the entire muscle, providing a protective covering and connecting it to surrounding structures. In certain areas, the epimysium thickens to form aponeurotic connective tissues, such as the erector spinae aponeurosis (IFAA ID: A04.3.02.003), which are known for their unique functions and mechanical properties, distinct from the typical epimysium [34]. The perimysium (IFAA ID: A04.0.00.042) is located within the muscle and divides it into bundles of muscle fibers, called fascicles (IFAA ID: A14.1.00.012). It acts as a supportive framework, enclosing and supporting these fascicles. Inside each fascicle, the endomysium (IFAA ID: A04.0.00.043) is present, surrounding individual muscle fibers and facilitating their proper functioning. At the ends of skeletal muscles, the aponeuroses, together with the epimysium, merge to form the tendon (IFAA ID: A04.0.00.044) [35]. Tendons are strong, fibrous structures that attach the muscle to the bone, transmitting the force generated by muscle contractions, allowing tissue strains and joint movement [35]. Histological studies have revealed that the endomysium, perimysium, epimysium, and tendons of skeletal muscles are tightly interconnected, for efficient force transmission from intermuscular to epimuscular tissues via the tendon, epimysium, and extramuscular connective tissue linkages [35,36,37]. 

The deep fascia and muscles are densely innervated. A study analyzed the thoracolumbar fascia and epimysial gluteal fascia in adult mice using transmission electron microscopy and immunohistochemistry, revealing a dense network of nerves within these fascial tissues, with significant differences in innervation between the two types of fascia [34]. In addition, skeletal muscles are provided by motor units and proprioceptors including muscle spindles and Golgi tendon organs, predominantly located near the myofascial elements within the muscle and adjacent to the deep fascia [34,38]. This suggests that myofascial force transmission potentially affects proprioception by transmitting muscle-generated forces through the fascial network, while sensory receptor cells in the fascia contribute to the body’s awareness of its position and movement. However, this remains a topic of debate, and further research is needed to confirm this.

### 2.4. Arthrofascia

The deepest fascia in the ArthroMyoFascial complex, referred to as arthrofascia, pertains to the fibrous connective tissue connections between bones that constitute a joint. The arthrofascia consists of capsules, ligaments, periosteum, and cartilage fibers [39]. The arthrofascial connections can be described as either segmental, involving two bones, or regional, involving three or more bones. The arthrofascia strongly determines passive joint motion [40]. In general, there are three types of arthrofascial connections for describing joint mechanics (synovial capsules, ligamentous, and via cartilage fibers). The synovial capsules (IFAA ID: A03.0.00.026) are dense fibrous connective tissues that merge with the periosteum and are attached at the ends of each of the involved bones forming a segmental joint. In certain areas, the capsule thickens to create capsular ligaments (IFAA ID: A03.0.00.036), which might also integrate tendons and other types of connective tissues [41]. In addition, ligaments (IFAA ID: A03.0.00.034) can also form a joint [42]. Compared to capsules, ligamentous connections regionally connect bones that work together to achieve specific functions. At last, bone linkages are formed by cartilage fibers, such as the annulus fibers of the discus (IFAA ID: A03.2.02.004) and ligamentum calcaneonaviculare plantare (IFAA ID: A03.6.10.203) [9,43]. All these anatomical structures, capsules, ligaments, and tendons attach to the periosteum via Sharpey’s fibers, which merge the periosteum (IFAA ID: A02.0.00.007) with the underlying bone tissue [44]. Consequently, we have classified it as arthrofascia, similar to how fascia from the muscles can be classified as myofascia. 

## 3. ArthroMyoFascial Complex: Proposed Concept for Clinicians

**Figure 1:**
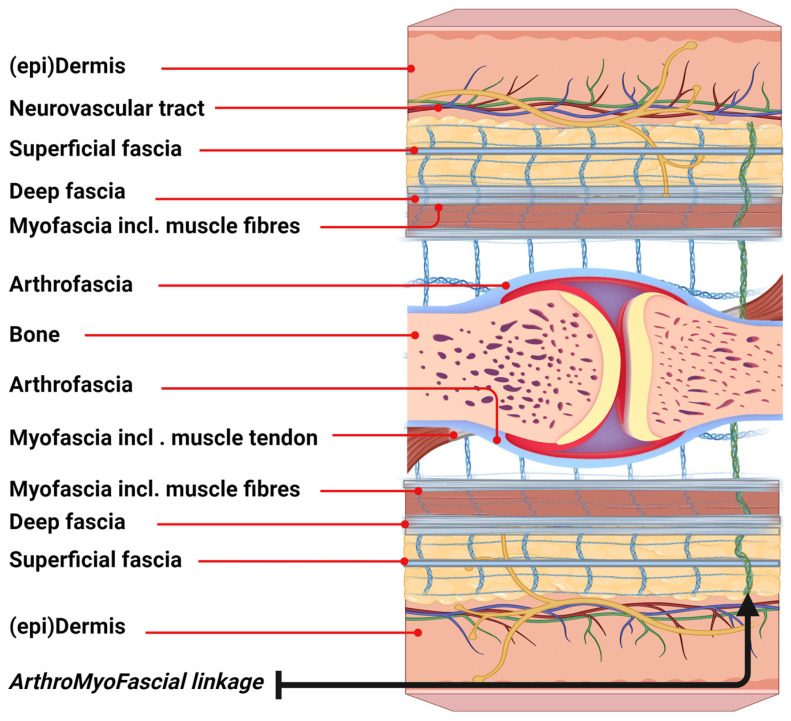
Schematic representation of the ArthroMyoFascial complex. This concept figure illustrates the interconnectivity of the ArthroMyoFascial complex encompassing synovial joint and skeletal muscles, crossing from the skin to the bone and from the bone back to the skin. Created with BioRender.com.

The ArthroMyoFascial complex (Figure 1) represents a comprehensive and integrated concept in basic fascia anatomy, highlighting the interconnection of various anatomical structures surrounding a joint in both humans and animals. This model clarifies the continuity of fascia at the joint level. From superficial to deeper layers, the model delineates the skin, including the (epi)dermis and superficial fascia, followed by the deep fascia, which overlies the myofascia and skeletal muscles. In addition, the model illustrates lateral connections with the arthrofascia, as well as the muscle–tendon linkage with the arthrofascia. Subsequently, the model demonstrates the inverse connections of deeper anatomical structures underneath the joint. Moreover, the connective tissue linkages depict the entire network in which these anatomical structures are embedded. It is important to note that the neurovascular tract also links these anatomical structures, although it is not fully represented in this model for clarity. This model could help expand our understanding of the myofascial unit, which plays a key role within the larger ArthroMyoFascial complex.

## 4. Proof of Concept: Ultrasound-Based Evaluation

To analyze the linkages between the skin, superficial fascia, deep fascia, and arthrofascia, we assessed the visualization of anatomical structures and linkages of the shoulder (glenohumeral), elbow (humeroradial), and thoracolumbar spine (spinal processes T12-L1) using 2D ultrasound imaging analysis. The 2D ultrasound images were obtained from the right and left side of two male subjects (subject 1: age 41 years, 178 cm, 78 kg, BMI 24.5; subject 2: age 24 years, 180 cm, 73 kg, BMI 22.2).

**Figure 2:**
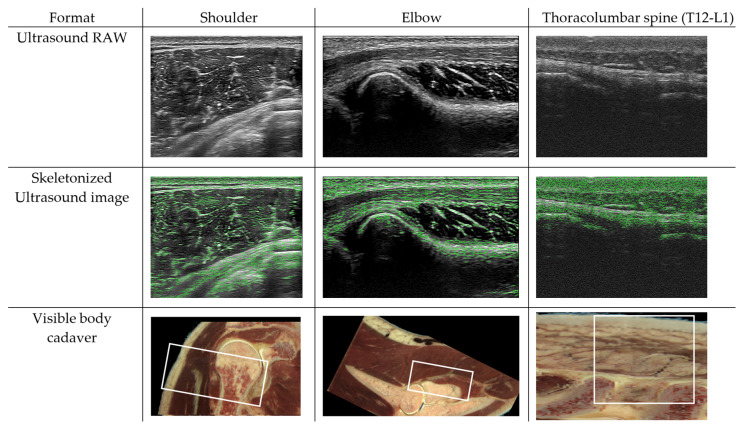
Skeletonized ultrasound images: proof of concept of ArthroMyoFascial linkages. Figure 2 represents images from the right shoulder and elbow as well as the spine. The first row depicts the raw images, which are non-processed images. The second row shows the skeletonized ultrasound images, revealing the connective tissue linkages from skin to bone. The last row represents approximately the same location as in a male human cadaver [48].

Ultrasound observations were conducted by the 2nd author using a Hitachi Arietta 850 SE, manufactured by Hitachi Ltd., Tokyo, Japan. Measurements were taken with a linear array probe (L64, 5–18 MHz) and ultrasonic gel (Aquasonic 100 Ultrasonic Gel, Parker Laboratories, Inc., Fairfield, NJ, USA). The feedback radio frequency of this signal was displayed in a two-dimensional ultrasound image (3 × 4 cm). The utilized gain (display value percentage) ranged between 55% and 80%, depending on the individual. The ultrasound images were analyzed in ImageJ (National Institutes of Health) to visualize the ArthroMyoFascial linkages [44]. First, the original image was opened and filtered using the Laplacian filter; subsequently, it was duplicated. The duplicate image was then converted to a binary image and subsequently skeletonized with the skeletonize function. The skeletonized lines, representing the connective tissue linkages, were colored, copied, and overlaid on the filtered ultrasound image. The skeletonized ultrasound images revealed a connective tissue matrix for all three locations (Figure 2). The skeletonization was performed 3 times by the 2nd author (ICC = 0.994). The validity of analyzing tissue networks was ensured by skeletonized cells and original photomicrographs [45]. Skeletonization is widely used in morphological analyses of biological structures, such as blood vessels, bony matrix, cell membranes, and more. It is useful for simplifying complex shapes and conducting quantitative and qualitative analyses [46,47]. However, the quality of the skeletonization method used to analyze connective tissue linkages in ultrasound images should be further tested.

The connective tissue linkages that were identified in our initial ultrasound analysis are important proof of concept. These linkages between the joints, muscles, and fascial systems via the applied evaluation techniques might be an important frontier of musculoskeletal research and therapy. To fully validate these findings, more research is needed, and research is warranted.

## 5. Discussion and Clinical Consideration

**Figure 3:**
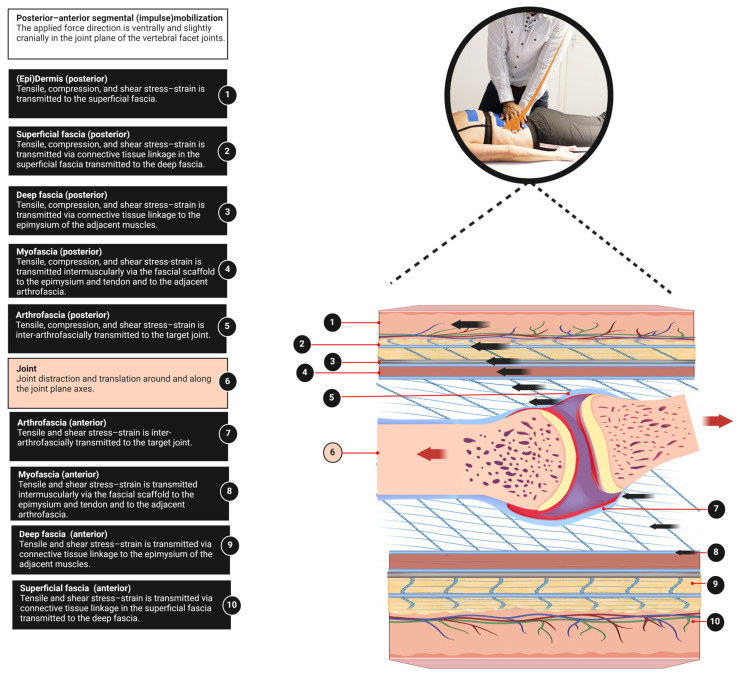
The skin as a force transmission pathway: This model represents how forces from the skin to the joint are transmitted during manual therapy. This applies to manual therapeutic interventions involving the skin, such as myofascial and joint mobilization techniques. Please note that the image does not depict anatomical accuracy for the lumbar spine, but rather a simplified representation to aid in understanding the concept. In this image, a posterior–anterior joint (impulse) mobilization with a cranial component is represented. This refers to a manual therapy approach used by healthcare practitioners, such as physiotherapists, osteopaths, and chiropractors, to address joint dysfunction or stiffness. The technique involves applying controlled pressure or an ‘impulse’ to the joint through the skin and underlying anatomical structures, moving from a posterior (back) to anterior (front) direction, and includes a cranial (head) component. It is important to note that for fascia tissue manipulations in general, the stress experienced and the displacement/strain is greater superior which reduces in depth (bullets 1:10). The main point we are addressing is that during joint mobilizations, the manipulative or mobilizing effects not only affect the arthrofascia but also influence the more superficial anatomical structures. This principle similarly applies to myofascial manipulations. In this figure, we visualize a cranial traction gapping component. While there are multiple components involved (traction, translation, rotation), for didactic purposes, we describe only the traction component. This method aims to apply a controlled pulling force that separates adjacent bones (traction), thereby increasing the joint space (creating a gapping effect) to stretch and soften the arthrofascia. Black vectors indicate the shear direction. Red vectors indicate the joint traction direction, also known as gapping manipulation/mobilization. Orange vector indicates the initial (impulse) mobilization direction applied by the manual therapist. Created with BioRender.com.

This perspective provides a basic anatomical model for clinicians to better understand fascia anatomy to complete the muscle–bone concept. An integral aspect of the ArthroMyoFascial complex is the recognition of a force-transmitting pathway between anatomical structures within this complex. In the ArthroMyoFascial complex, the force-transmitting pathway refers to the continuous network of connective tissue linkages from superficial to deeper layers and vice versa. However, it should be acknowledged that our model does not fully explain the longitudinal intermuscular force-transmitting pathways. These pathways require other anatomical explanatory models, such as the myofascial trains [31,49]. It is worth noting that these myofascial models typically do not encompass the skin, superficial fascia, and arthrofascia, which are covered in our ArthroMyoFascial model. This implies that passive stiffness surrounding a myofascial unit may arise not only by its myofascia but as well from more superficially located fascial tissues such as the superficial fascia and deep fascia, as well as from deeper structures like the arthrofascia of joints. Additionally, understanding this concept may assist in elucidating the mechanism of force transmission from the skin to the bone and vice versa. For instance, it can explain how displacement of the skin results in the transmission of stress to anatomical structures beneath the skin (Figure 3). This might be a key effect of various manual therapeutic practices, such as joint (impulse) mobilizations, myofascial manipulations, and elastic tape application, where force is transmitted from the skin to the superficial fascia, deep fascia, myofascia, and the arthrofascia (Figure 3). Furthermore, it may contribute to the clinical understanding of the interaction between joints, myofascia, and deep fascia. For instance, during secondary hip traction, the applied force stresses the arthrofascia but also the deep fascia and myofascia to a lesser extent [13,50]. It is significant that changes in relative stiffness alter the stress transmission pathways between fascia, muscles, and joints [13]. The ArthroMyoFascial complex can enhance clinical understanding by highlighting how increased epi- and extramuscular connective tissues may disrupt the force generated by the myofascial unit. In addition, it suggests that stiffness in the superficial fascia could hinder access to deeper layers of myofascia and/or arthrofascia during joint mobilizations or manipulations. For example, softening the superficially stiffened fascial tissues through myofascial release may be crucial for achieving the desired therapeutic effects of other fascial tissue manipulations, such as elastic tape application and joint mobilizations/manipulations. However, we want to emphasize that this is an anatomical model specifically designed for clinical therapists, and while it does not represent the complete anatomical truth, it provides a useful framework for understanding and approaching complex mechanical processes.

## 6. Conclusions

The ArthroMyoFascial complex consists of multiple layers from superficial to deeper anatomical structures, namely the skin, superficial fascia, deep fascia, myofascia including muscle fibers, and arthrofascia, all linked within a connective tissue matrix. This model indicates that it is a force-transmitting system from the skin to the bone. This information is essential for manual therapists, including physiotherapists, osteopaths, chiropractors, and massage therapists, as they all work with fascial tissues within the musculoskeletal domain. Adopting this basic approach enables clinicians to effectively address various body anatomical structures, enhancing the clinical understanding of fascia within the muscle–bone concept. This is essential for therapeutic testing, treatment, reporting, and multidisciplinary communication, so is important for comprehending musculoskeletal and orthopedic rehabilitation.

## Figures and Tables

**Table 1:** ArthroMyoFascial anatomical structures, a subdivision.

Superficial Fascia	Deep Fascia	Myofascia	Arthrofascia
Fascia superficialis (A04.0.00.031)	Fascia profunda (A04.0.00.031)	Fascia musculorum (A04.0.00.038)	Retinaculum (e.g., Retinaculum flexorum) (A04.7.03.026)
Retinacula cutis fibers superior (A16.0.00.005)	Thoracolumbar fascia (A04.3.02.501)	Epimysium (A04.0.00.041)	Ligaments (A03.0.00.034)
Superficial fascial membrane (A04.0.00.031)	Lateral Raphe (A04.3.02.501)	Perimysium (A04.0.00.042)	Capsular ligaments (A03.0.00.036)
Retinacula cutis fibers inferior (A16.0.00.005)	Fascia lata (A04.7.03.002)	Endomysium (A04.0.00.043)	Capsules (A03.0.00.026)
Skin ligaments (Cooper’s ligaments) (A16.0.00.005)	Muscular septi (e.g., brachial intermuscular septa) (A04.6.03.006)	Muscle fascicle (A14.1.00.012)	Periosteum (A02.0.00.007)
Camper’s fascia (abdomen) (A16.0.03.002)	Popliteal fascia (A01.2.08.013)	Myotendinous junction	Annulus fibers (A03.2.02.004)
	Iliotibial tract (A04.7.03.003)	Erector spinae aponeurosis (A04.3.02.003)	Sharpey’s fibers (insertion tendon vessels-periosteum)
	Deep cervical fascia (alar fascia)		
	Fascia cruris (A04.7.03.021)		
	Plantar fascia (foot) (A04.7.03.031)		
	Scarpa’s fascia (abdomen) (A04.5.02.022)		

This table provides a brief overview/subdivision of anatomical fascial structures including their anatomical identifier codes. The first row indicates the depth of the structures relative to the (epi)dermis in column 1:4 (superficial fascia, deep fascia, myofascia, and arthrofascia, respectively). The following rows most often indicate their location in the body. Not all anatomical fascial structures are included in this table, but it offers a concise impression of this anatomical subdivision. Note that the ArthroMyoFascial complex includes neurovascular tracts and lymphatic vessels, which also interconnect all tissues.

## Data Availability

Not applicable.

## References

[1] Swart N.M., Apeldoorn A.T., Conijn D., Meerhoff G.A., Ostelo R. (2021). KNGF Clinical Practice Guideline for Low back pain and lumbosacral radicular syndrome. KNGF-Richtlijn.

[2] Apeldoorn A.T., Swart N.M., Conijn D., Meerhoff G.A., Ostelo R.W. (2024). Management of low back pain and lumbosacral radicular syndrome: The Guideline of the Royal Dutch Society for Physical Therapy (KNGF). Eur. J. Phys. Rehabil. Med..

[3] Stecco C., Day J.A. (2010). The fascial manipulation technique and its biomechanical model: A guide to the human fascial system. Int. J. Ther. Massage Bodyw..

[4] Starich M. (2017). Rolfing^®^ Structural Integration. Integrative Geriatric Medicine.

[5] Kase K. (2008). Clinical Therapeutic Applications of the Kinesio (! R) Taping Method.

[6] Simmonds N., Miller P., Gemmell H. (2012). A theoretical framework for the role of fascia in manual therapy. J. Bodyw. Mov. Ther..

[7] Amstel R.v., Noten K., Malone S., Vaes P. (2023). Fascia Tissue Manipulations in Chronic Low Back Pain: A Pragmatic Comparative Randomized Clinical Trial of the 4xT Method^®^ and Exercise Therapy. Life.

[8] Amstel R.N.v., Jaspers R.T., Pool-Goudzwaard A.L. (2022). Skin Displacement as fascia tissue manipulation at the lower back affects instantaneously the flexion-and extension spine, pelvis, and hip range of motion. Front. Physiol..

[9] Schleip R., Jäger H., Klingler W. (2012). What is ‘fascia’? A review of different nomenclatures. J. Bodyw. Mov. Ther..

[10] Schleip R., Hedley G., Yucesoy C.A. (2019). Fascial nomenclature: Update on related consensus process. Clin. Anat..

[11] Herrmann M., Engelke K., Ebert R., Müller-Deubert S., Rudert M., Ziouti F., Jundt F., Felsenberg D., Jakob F. (2020). Interactions between muscle and bone—Where physics meets biology. Biomolecules.

[12] Avin K.G., Bloomfield S.A., Gross T.S., Warden S.J. (2015). Biomechanical aspects of the muscle-bone interaction. Curr. Osteoporos. Rep..

[13] Maas H. (2019). Significance of epimuscular myofascial force transmission under passive muscle conditions. J. Appl. Physiol..

[14] Stecco L. (2002). Manipolazione Della Fascia: Per il Trattamento Delle Affezioni Muscoloscheletriche.

[15] Stecco A., Giordani F., Fede C., Pirri C., De Caro R., Stecco C. (2023). From Muscle to the Myofascial Unit: Current Evidence and Future Perspectives. Int. J. Mol. Sci..

[16] Huijing P.A. (2009). Epimuscular myofascial force transmission: A historical review and implications for new research. International Society of Biomechanics Muybridge Award Lecture, Taipei, 2007. J. Biomech..

[17] Adstrum S., Hedley G., Schleip R., Stecco C., Yucesoy C.A. (2017). Defining the fascial system. J. Bodyw. Mov. Ther..

[18] Schleip R., Gabbiani G., Wilke J., Naylor I., Hinz B., Zorn A., Jäger H., Breul R., Schreiner S., Klingler W. (2019). Fascia is able to actively contract and may thereby influence musculoskeletal dynamics: A histochemical and mechanographic investigation. Front. Physiol..

[19] Stecco C., Fede C., Macchi V., Porzionato A., Petrelli L., Biz C., Stern R., De Caro R. (2018). The fasciacytes: A new cell devoted to fascial gliding regulation. Clin. Anat..

[20] Junqueira L.C.U., Carneiro J. (2005). Basic Histology: Text & Atlas.

[21] Ferreira L.M., Hochman B., Locali R.F., Rosa-Oliveira L.M. (2006). A stratigraphic approach to the superficial musculoaponeurotic system and its anatomic correlation with the superficial fascia. Aesthetic Plast. Surg..

[22] Clarys J., Provyn S., Marfell-Jones M. (2005). Cadaver studies and their impact on the understanding of human adiposity. Ergonomics.

[23] Herlin C., Chica-Rosa A., Subsol G., Gilles B., Macri F., Beregi J.P., Captier G. (2015). Three-dimensional study of the skin/subcutaneous complex using in vivo whole body 3T MRI: Review of the literature and confirmation of a generic pattern of organization. Surg. Radiol. Anat..

[24] Hedley G. Interstitium? Perifascia, Aka the Fuzz! (Video for 2018 Fascia Congress, Berlin). https://www.gilhedley.com/clips.

[25] Fede C., Petrelli L., Pirri C., Neuhuber W., Tiengo C., Biz C., De Caro R., Schleip R., Stecco C. (2022). Innervation of human superficial fascia. Front. Neuroanat..

[26] Stecco C., Porzionato A., Lancerotto L., Stecco A., Macchi V., Day J.A., De Caro R. (2008). Histological study of the deep fasciae of the limbs. J. Bodyw. Mov. Ther..

[27] Stecco C., Gagey O., Belloni A., Pozzuoli A., Porzionato A., Macchi V., Aldegheri R., De Caro R., Delmas V. (2007). Anatomy of the deep fascia of the upper limb. Second part: Study of innervation. Morphologie.

[28] Vleeming A., Schuenke M., Danneels L., Willard F. (2014). The functional coupling of the deep abdominal and paraspinal muscles: The effects of simulated paraspinal muscle contraction on force transfer to the middle and posterior layer of the thoracolumbar fascia. J. Anat..

[29] Benjamin M. (2009). The fascia of the limbs and back—A review. J. Anat..

[30] Willard F., Vleeming A., Schuenke M., Danneels L., Schleip R. (2012). The thoracolumbar fascia: Anatomy, function and clinical considerations. J. Anat..

[31] Krause F., Wilke J., Vogt L., Banzer W. (2016). Intermuscular force transmission along myofascial chains: A systematic review. J. Anat..

[32] Schuenke M., Vleeming A., Van Hoof T., Willard F. (2012). A description of the lumbar interfascial triangle and its relation with the lateral raphe: Anatomical constituents of load transfer through the lateral margin of the thoracolumbar fascia. J. Anat..

[33] Bogduk N., Macintosh J.E. (1984). The applied anatomy of the thoracolumbar fascia. Spine.

[34] Fede C., Petrelli L., Guidolin D., Porzionato A., Pirri C., Fan C., De Caro R., Stecco C. (2021). Evidence of a new hidden neural network into deep fasciae. Sci. Rep..

[35] Purslow P.P. (2020). The structure and role of intramuscular connective tissue in muscle function. Front. Physiol..

[36] Sensini A., Massafra G., Gotti C., Zucchelli A., Cristofolini L. (2021). Tissue engineering for the insertions of tendons and ligaments: An overview of electrospun biomaterials and structures. Front. Bioeng. Biotechnol..

[37] Huijing P. (2008). Intramuscular myofascial force transmission. Encyclopedia of Neuroscience.

[38] James G., Stecco C., Blomster L., Hall L., Schmid A.B., Shu C.C., Little C.B., Melrose J., Hodges P.W. (2022). Muscle spindles of the multifidus muscle undergo structural change after intervertebral disc degeneration. Eur. Spine J..

[39] Noten K., Amstel R.N.v. Introducing the Arthro-Myofascial Complex. (4xT®Method the ArthroMyofascial Therapy: Chapter 3)1. Fascia More than Myofascia!. https://osf.io/d85k3/wiki/ArthroMyofascial%20Complex/.

[40] Widmer J., Cornaz F., Scheibler G., Spirig J.M., Snedeker J.G., Farshad M. (2020). Biomechanical contribution of spinal structures to stability of the lumbar spine—Novel biomechanical insights. Spine J..

[41] Martin H.D., Savage A., Braly B.A., Palmer I.J., Beall D.P., Kelly B. (2008). The function of the hip capsular ligaments: A quantitative report. Arthrosc. J. Arthrosc. Relat. Surg..

[42] Bejarano-Pineda L., Guss D., Waryasz G., DiGiovanni C.W., Kwon J.Y. (2021). The Syndesmosis, Part I: Anatomy, Injury Mechanism, Classification, and Diagnosis. Orthop. Clin..

[43] Bartoníček J., Rammelt S., Naňka O. (2018). Anatomy of the subtalar joint. Foot Ankle Clin..

[44] Petermann H., Sander M. (2013). Histological evidence for muscle insertion in extant amniote femora: Implications for muscle reconstruction in fossils. J. Anat..

[45] Young K., Morrison H. (2018). Quantifying microglia morphology from photomicrographs of immunohistochemistry prepared tissue using ImageJ. JoVE (J. Vis. Exp.).

[46] Stevens C.R., Berenson J., Sledziona M., Moore T.P., Dong L., Cheetham J. (2020). Approach for semi-automated measurement of fiber diameter in murine and canine skeletal muscle. PLoS ONE.

[47] Marks P.C., Preda M., Henderson T., Liaw L., Lindner V., Friesel R.E., Pinz I.M. (2013). Interactive 3D analysis of blood vessel trees and collateral vessel volumes in magnetic resonance angiograms in the mouse ischemic hindlimb model. Open Med. Imaging J..

[48] NIH, National Institute of Health (2013). The Visible Human Project.

[49] Myers T.W. (2013). Anatomy Trains E-Book: Myofascial Meridians for Manual and Movement Therapists.

[50] Estébanez-de-Miguel E., López-de-Celis C., Caudevilla-Polo S., González-Rueda V., Bueno-Gracia E., Pérez-Bellmunt A. (2020). The effect of high, medium and low mobilization forces applied during a hip long-axis distraction mobilization on the strain on the inferior ilio-femoral ligament and psoas muscle: A cadaveric study. Musculoskelet. Sci. Pract..

